# “We don’t really know what else we can do”: Parent experiences when adolescent distress persists after the Maudsley and family-based therapies for anorexia nervosa

**DOI:** 10.1186/s40337-019-0235-5

**Published:** 2019-02-12

**Authors:** Ella Wufong, Paul Rhodes, Janet Conti

**Affiliations:** Western Sydney University, Locked Bag 1797, Penrith, 2751 Australia

**Keywords:** Adolescent anorexia nervosa, Maudsley family therapy, Family-based therapy, Parent experiences, Identity, Qualitative, Discourse

## Abstract

**Background:**

Maudsley Family Therapy (MFT), and its manualised version, Family-Based Therapy (FBT), are the only well-established treatment interventions for adolescent anorexia nervosa (AN), with treatment efficacy primarily measured by improvements in eating behaviours and weight restoration. A crucial component of this therapy is an intensive home-based refeeding intervention that requires a substantial commitment from parents for up to one year. While this treatment works to restore weight in a proportion of adolescents, very little is known about its impacts on family distress, relationships and identity, including in the 40% of families where the adolescent experiences ongoing eating disorder (ED) symptomatology and/or psychological distress during and post-treatment. Specifically, few studies have investigated the impacts of MFT/FBT treatment on family functioning or on how parents negotiate their identities, or who they understand themselves to be, in the context of this treatment intervention. This is a significant omission, given the substantive role assigned to parents to take responsibility for their child’s eating restoration in the first treatment phase. This study seeks to address this gap through a qualitative exploration of parents’ experiences of MFT/FBT, in cases where treatment was discontinued and/or their child continued to experience psychological distress post-treatment.

**Methods:**

13 parents participated in in-depth semi-structured interviews that scaffolded between their experiences and ways they negotiated and sustained their identities as parents within the context of MFT/FBT for their child. Interview data was analysed through a framework of critical discursive analysis to generate themes centred on these parents’ experiences and identity negotiation.

**Results:**

Key findings are that MFT/FBT: (1) provided a map for therapy that initially relieved parents’ anxieties for their child and facilitated improvements in family functioning; (2) inadequately addressed parental guilt and blame with a form of externalisation of the illness; (3) perpetuated parental guilt by raising anxiety about AN and allocating responsibility for refeeding their child in phase 1 of the treatment; and (4) when ceased, left these parents struggling with an uncertain future, and fears for the wellbeing of their children.

**Conclusions:**

The structure of MFT/FBT provided initial relief with some improvements in family communication patterns, however, when the adolescent experienced protracted ED symptoms and/or ongoing psychological distress post-treatment, these parents were left with uncertainty as to how to navigate their shifting roles and their child’s ongoing struggles. This research highlights the need for treatments for adolescent AN that more comprehensively address both the adolescent and parents’ psychological distress and also (re)build their senses of identity that have been challenged by AN and its effects.

## Plain English summary

The Maudsley model of family therapy for anorexia nervosa is a well-established intensive approach to the treatment of children and teens. It requires a substantial commitment from parents who must engage in intensive home-based refeeding and therapy for up to one year. Parents are encouraged to dedicate much of their lives to this process, with siblings also providing emotional support to the adolescent. While this treatment works for many very little is known about the effects it has on family distress, relationships and identity. Little is also known about these effects for families who it does not work for. In this project we interviewed thirteen families to find out more. Many described how the treatment did provide them with a road map that helped make them feel less anxious and restore family relationships for a while. In the long term, however, some described how the treatment could make them feel more guilty when it didn’t work as well as had been promised and make them anxious about the future for their child.

## Background

Maudsley Family Therapy (MFT), and its manualisation into Family-Based Treatment (FBT) are the most frequently researched treatment interventions for adolescent anorexia nervosa (AN), with reported long-term improvements in adolescent eating behaviour and weight restoration. This has led to these interventions meeting the American Psychological Society’s (APS) “well established treatment” (level 1) criteria [1] and inclusion in the NICE treatment guidelines [2]. In the growing body of research on MFT and FBT, including treatment augmentations, there is a paucity of in-depth analysis of the experiences of parents whose adolescents and family are treated with MFT/FBT for adolescent AN. In particular there is an absence of the voice of the 40% of parents whose adolescents experience ongoing psychological distress and/or ED symptomatology post-treatment, despite weight restoration [3].

MFT was developed by clinicians and researchers at the Maudsley Hospital in London [4, 5] and later manualised by Lock and Le Grange [6, 7] into FBT [7]. MFT/FBT^1^ is a pragmatic synthesis of a number of other family therapy approaches that have been used to treat AN [7]. The intervention borrows the “family meal” and allocating responsibility to the parents for their child’s nutritional restoration from Minuchin’s structural family therapy approach [8], the agnostic view of etiology from strategic family therapy [9, 10], therapist neutrality in relation to the family system from Milan systems therapy [11, 12], and the externalisation of the illness from Narrative Therapy (NT) [13]. A key feature of the approach is that it seeks to harness the parents (and siblings) as the fundamental resources in the “fight” against “the anorexia” ([7], p. 80). The first phase of treatment is focused exclusively on supporting the parents to assume control over their child’s food intake, which continues until the adolescent has reached at least 90% of their expected body weight ([7], p. 179). The second phase of treatment focuses on transitioning control of eating back to the adolescent, and the final phase focuses on the adolescent’s developmental needs.

With the measure of remission from AN as ≥95% Ideal Body Weight (IBW), less than half of adolescents who undergo FBT recover [14, 15], and a randomized controlled trial (RCT) of long-term outcomes of adolescents treated with FBT reported that although 89% of adolescents achieved weight gain to 90% IBW, around 40% of these adolescents had significant ongoing psychological distress at follow-up, as reported by them or their parents [3]. Furthermore, drop-out rates of approximately 20% have been reported for MFT/FBT interventions [16, 17]. Although argued by some to be the “gold-standard” treatment for AN [18] the effectiveness of this intervention is uncertain for a notable proportion of adolescents, outside the outcome measure of weight restoration.

Since its development, there has been a number of research studies that have augmented components of the MFT and FBT interventions. Variations on the delivery of these interventions have included treatment of the adolescent separated from the parents (parent-focused therapy), systemic family therapy and multi-family group interventions. These variations have been rated by the APS criteria as “possibly efficacious” (level 3) [1]. Further augmentations to the manualised FBT approach [19] have proliferated and include the addition of individual Cognitive Behaviour Therapy to target perfectionism [20], incorporation of the principles of exposure therapy [21], and Dialectical Behaviour Therapy [22]. A recent systematic review [23] has reported that while some of these augmentations focus on the young person’s psychological issues, the majority increase the intensity of parent-driven behavioural intervention through multi-family therapy [24, 25], separated family therapy [26, 27], parent-focused therapy (PFT) [15], intensive family coaching [28] and day hospital permutations [29]. As high levels of expressed emotion (EE) and parental criticism have been hypothesised as possible impediments in the standard conjoint FBT model [30, 31], PFT avoids this issue by separating the adolescent from the parent in therapy and has been found to have improved treatment outcomes in families reporting high expressed emotion [26]. Notably evident in the majority of innovations in family interventions for adolescent AN is a reluctance to question the core principles of the original Maudsley model [32], or to include therapeutic practices that are seen as corrupting those principles.

Despite a focus on MFT/FBT augmentations, evidence for their benefit is not robust [1]. Most importantly there is no strong evidence to suggest how the manualised FBT model might be augmented to make it more responsive to the specific needs of a family struggling with MFT/FBT. A more responsive model has been developed by researchers and clinicians associated with the Maudsley Hospital in London—the “New Maudsley Approach” [33] aims to provide improved collaborative support and skills training for parents and incorporates elements of Motivational Interviewing [34] into its collaborative skills training program to address the treatment ambivalence that is often a feature of anorexia presentations [35]. The New Maudsley Approach is grounded in the belief that parents of children with AN are already experiencing high levels of stress and distress, and therefore interventions that intentionally raise anxiety levels—such as the orchestration of the “intense scene” in the first session of FBT ([7], p. 59) may ultimately prove unhelpful.

In the evidence base for the outcomes of MFT/FBT there is a notable omission of parental measures [36] and a paucity of in-depth research into parents’ experiences and ways they negotiate the structure and components of the intervention. A metasynthesis of adolescents, parents, and clinicians who treat adolescent AN [37] has highlighted a dissonance between treatment “targets”; for example, the priority for clinicians was found to be AN and its eradication through focusing on weight gain and behaviour change, whereas adolescents were consistently found to be concerned with psychological and social functioning [38]. Parents also expected treatment targets to extend beyond a somatic focus and the authors argued that treatment that is overly focused on weight and behaviour change risks creating “pseudorecovery” ([37], p. 14) that is, physical recovery in the absence of psychological recovery, with a high risk of relapse. The issue of treatment targets has been echoed in several studies where adolescents expressed the view that an overemphasis on physical attributes rather than psychological factors was unhelpful and dehumanising [39–41]. From a parental perspective in another qualitative study, two fathers also expressed frustration at the emphasis on weight gain [42]. Likewise, a follow-up study of parents and adolescents at one-year follow-up after an FBT intervention found that a significant minority of participants reported an overemphasis on eating behaviours, and insufficient attention paid to causative factors and psychological issues [43].

This current study is an in-depth analysis of the experiences of 13 Australian parents who experienced MFT/FBT for AN treatment for their child and either dropped-out from this treatment or reported that their child continued to experience psychological distress and/or ED symptomatology, despite partial or full weight restoration. The aims of this research were, firstly, to build a more comprehensive understanding of how these parents ascribed meaning to their experiences and negotiated their identities in the context of MFT/FBT, which is important given the central role parents are assigned in this intervention to facilitate their child’s weight and eating recovery. Secondly, this study sought to establish a platform to inform the development of future treatment interventions for adolescents who experience AN and their families.

## Methods

### Participants

Thirteen parents (nine mothers and four fathers) of 11 female adolescents were interviewed about their experiences of MFT/FBT intervention after their daughter’s diagnosis of AN (see Table 1 for details). Although eight of the 11 adolescents had returned to within a normal weight range with MFT/FBT, one had lapsed into low body weight, and all the adolescents experienced ongoing psychological distress (reported by the parent and/or adolescent). Twelve of the 13 parents were living with the other birth parent of their children. Eleven of the parents participated in this study between 1 and 6 years post-MFT/FBT, and two parents reported MFT for their daughter being 15 years prior. All three adolescents who discontinued MBT/FBT before weight restoration continued to engage in individual therapy, with one subsequently reaching restoration to a normal weight. Three of the 11 adolescents were permitted to engage in individual therapy during MFT/FBT (see Table 2 for details of participants’ AN treatments).

**Table 1:** MFT/FBT treatment and reported adolescent outcome

Parent Pseudonyms	Family structure	Child age at interview	Child diagnosis age	MFT/ FBT age	MFT/ FBT mths	Individual therapy during MFT/FBT	Phase completed	Adolescent symptoms after MFT/FBT
Kristin & Nathan	Parents +3C	28	13	13	12	No	3	WR, ED, D (P, A)
Alice	Parents +2C	14	12	12	10	Yes. OCD	2 (current)	WR, ED, D (P)
Emily	Parents +2C	18	16	16	19	Yes. Depression & anxiety	2	WR, ED, D (P)
Jane	Parents +3C (NZ)	20	16	16	6	No	3	WR, ED, D (P, A)
Janice	Parents +2C	20	14	14	24	Yes. CBT, mindfulness, psychiatrist	3	WR, ED, D (P, A)
Kiera	Parents +3C	20	14	14	30	No	3	WR, ED (lapse after 3 years) & D (P)
Paul	Parents +4C	19	16	16	24	Yes. Depression & anxiety	3	WR, ED, D (recent hospitalisation) (P, A)
Terry	Parents +2C	18	14	14	6	No. Psychologists & psychiatrists before & after	2	WR, lapse into ED (low weight), D (P, A)
Margaret & Jack	Parents +2C	14	11	11	24	No. Until end of MFT/FBT	1 (DC)	Not WR, ED, D (gained weight with other ED treatment) (P, A)
Merrum	Parents separated +3C	18	17	17	3–4	No	1 (DC)	Not WR, ED, D (P, A)
Susan	Parents +2C	21	15	15	12–24	No. Psychologist when inpatient	1 (DC)	Not WR, ED, D (P, A)

Abbreviations: *C* child, *DC* discontinued, *WR* weight restored, *ED* eating disorder, *D* psychological distress, *P* parent report, *A* adolescent report

**Table 2:** Adolescent treatments for ED and other psychological problems

Parent	Other eating disorder treatments	Eating disorder behaviours	Treatment for other problems
Kristin & Nathan	Psychiatrist, BN-Day program, DBT group (all C)	Current binge eating started 2 years after MFT/FBT	Psychiatrist and DBT group (C) for depression, self-harm
Alice	Inpatient (P)	Restriction, over-exercise	Psychiatrist (C) and inpatient (P), OCD (diagnosed at 11 years old), depression, self-harm, possible ASD
Emily	Inpatient (including nasogastric tube), dietician (P)	Restriction, over-exercise	Psychologist from age 14 for anxiety, depression, self-harm
Jane	Inpatient, (P) group, (C), psychologists (C & P)	Restriction, over-exercise, purging, binging	Depression, sub-clinical ASD symptoms (diagnosed at 10–12 years)
Janice	Psychologist (CBT & mindfulness), ED support group (C & P), psychiatrist (C)	Restriction, over-exercise, vomited once	Psychiatrist (C), OCD/depression currently on lithium and recently ceased escitalopram
Kiera	Nil. Thinking of seeing a psychologist	Restriction and over-exercise	Psychiatrist, Psychologists (P), anti-depressants (subsequent to anorexia diagnosis), self-harm
Paul	3 inpatient admissions (P), about to recommence hospital treatment	Restriction, over-exercise, purging and binge eating	Psychiatrists/psychologists/school counsellor (P) for depression, anxiety (included anti-depressants)
Terry	Inpatient then MFT/FBT (P), outpatient, multi-family therapy (P)	Restriction, over-exercise, purging	Psychiatrist/psychologist (C), self-harm, Conversion Disorder, depression, psychosis: medication including quetiapine
Margaret & Jack	Psychologist (NT) (C), inpatient admission (P)	Restriction, over-exercise	Psychologist (C), anxiety
Merrum	Psychologist (NT), dietitian, psychiatrist (C)	Restriction, over-exercise	Psychologist and Psychiatrist (C), anxiety, family counselling before ED (P)
Susan	Psychologist and psychiatrist (C), inpatient admissions (P)	Restriction, over-exercise, purging	Psychologist and psychiatrists for depression, OCD (C)

Abbreviations: *C* current, *P* past

### Procedure and materials

This study was approved by the Western Sydney University Human Research Ethics Committee (approval number: H11303). Participants responded to advertisements distributed through health professional networks, which invited them to participate in a research study with the question: “How can we improve Maudsley Family Therapy for Adolescent Anorexia?”

Semi-structured interviews were conducted in person, or via telephone. Three interviews were carried out by author (EW) in 2017, and the remainder by author (JC) and an additional researcher in 2016. The semi-structured interview schedule (Appendix A) allowed for unstructured questions that were guided by participant’s responses in vivo [44] and drew from the paradigm of NT [45] to scaffold between the parent’s experiences of the MFT/FBT intervention and ways that they negotiated their identities within this context. Analysis of these parent experiences, identity negotiations and dilemmas provided a foundation for ways MFT/FBT could be improved. The interviews were audio-recorded, and transcribed with the method of light transcription [46], where repetitive words, phrases and pauses (unless lengthy) that disrupt the flow and coherence of the accounts are removed, thereby enabling the analysis to track shifts in positioning and discursive resources used, rather than focusing on the more conversational elements of speaking. All names in the transcripts were replaced with participant-chosen pseudonyms, and participants were given a copy of the interview transcript to change and/or remove information that they considered potentially identifying. Participants were also given a copy of this paper to member-check the analysis of their transcripts as a form of validity (see Appendix B for parent review and feedback).

### Analysis

To address the research questions and develop a richer understanding of these parents’ experiences, patterns of identity negotiation, and dilemmas associated with key components of MFT/FBT, a critical discursive analysis framework [47, 48] was drawn upon to analyse the interview data. Analysis by the researchers (see Appendix C for researcher positioning statements) traced some of the discursive materials these parents used to piece together narratives of their experiences of MFT/FBT, including their active negotiation of dilemmas, ways they were positioned by and positioned themselves in relation to dominant discourses [49], and from these positions authored a unique sense of identity as parents. Initial coding of the interview data as relevant to the research questions was collapsed into a set of themes that were refined in a recursive process. Interview extracts that most richly represented the final set of themes and subthemes were then extracted and analysed in greater depth.

## Results

**Fig. 1:**
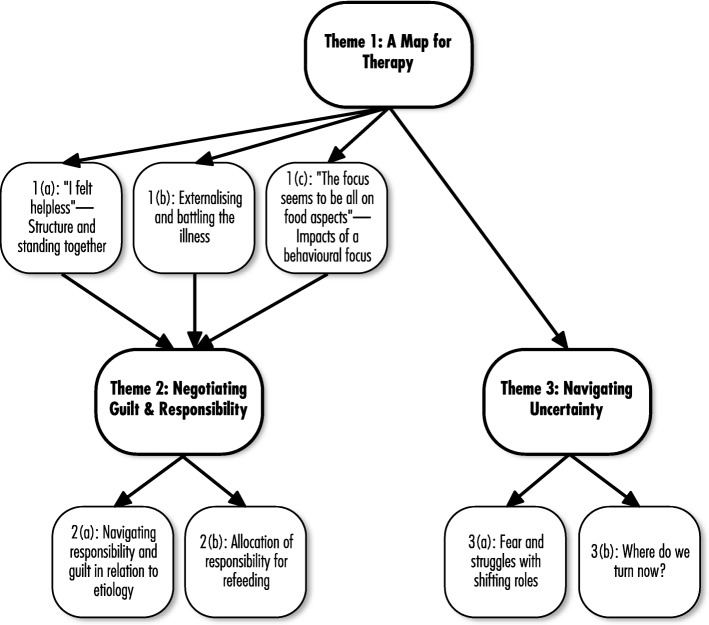
Thematic Map of Parents’ Experiences of MFT/FBT

Analysis traced these parents’ experiences of key dimensions of the MFT/FBT interventions and concludes with the uncertainty faced by parents after ceasing treatment (see thematic map, Fig. 1).

### Theme 1—A map for therapy

Although the 13 parents experienced initial relief through the MFT/FBT map, 11 were also troubled by the extent of the focus on their child’s eating restoration—frequently at the exclusion of addressing their child’s psychological distress—especially in the early phases of treatment.

#### “I felt helpless”: Structure and standing together

Nine parents talked about a sense of relief on finding professionals who specialised in AN treatment and the structured MFT/FBT interventions.

*Extracts 1*

*Emily: I think I was extremely grateful to the Maudsley team, […] the first people who actually talked to us, right, to explain things to us, who gave us a guideline.*

*Janice: I felt helpless. I felt totally out of control. I had lots of people saying to me “Just—just make her eat. It’s no big deal.” Particularly […] that generation who don’t always get that this is a genuine mental illness. So, having a plan just made me feel like I had something secure to—to work on, to work with, to trust in. And I trusted the psychiatrist we were seeing emphatically.*

*Jack: […] the 95% Maudsley track record or whatever it was that rolled out gave me a high level of confidence ‘will this work?’ So follow it, follow them, follow ah it consistently, don’t deviate because we haven’t got any of that expertise or experience they do.*

These narratives exemplify the extent of these parents’ pain and helplessness in the context of their child’s AN experience. Being given a “guideline” by the Maudsley treatment team was experienced as a relief by Emily after a paucity of explanations in previous treatments. Diagnosis for Janice legitimated her daughter’s experience as a “genuine mental illness”—and confirmed her insider knowledge of the complexity of her daughter’s experience—that was not simply attributable to parental incompetence in not being able to “just make her eat”. The high evidence of success of MFT/FBT cited to Jack contributed to an initial confidence in handing over of “expertise” of their daughter’s care, as AN had undermined his own expertise as her father.

The structure of MFT/FBT therapy also had the potential to assist in the restructuring of family relationships as outlined in nine of the parents’ narratives.

*Extracts 2*

*Emily: […] they actually kind of guided us through some of the very, very hard conversations. Hard as in about feelings, about guilt, about perceptions, and about the effects it had on us […] I think as a family we’ve grown a lot closer […] in a way that we can respect each other.*

*Nathan: […] the kids could complain about us, we could complain about them. So it, it developed an openness that hadn’t been there previously.*

*Kiera: […] we were a joint force. I think if you can work together really quickly it helps because an eating disorder can get around one of you, but if it knows that dad’s there backing up everything that mum says and vice-versa, I think that helps.*

*Jane: […] she announced that she appreciates what we did for her […] She knows we’re quite committed to it as best as we can.*

These extracts highlight the scope for MFT/FBT in cultivating a sense of closeness in relationship between family members. For Emily, MFT/FBT provided a scaffold for her family to engage in “very hard conversations” that navigated questions about “guilt” and “perceptions”. For other parents, these interventions provided a context for “openness” and for their child to have a voice (Nathan) and, through the structural family intervention, a unified voice to the parents (Kiera). MFT/FBT also provided opportunity for these parents to experience themselves as contributing positively and meaningfully to their children’s lives through their commitment to their child’s recovery.

#### Externalising and battling the illness

Nine parents talked specifically about their experiences of externalisation of the illness, including the process of separating their child’s identity from the illness identity, and fighting the illness, rather than their child.

*Extracts 3*

*Kiera: There was Sally and there was the eating disorder and once you separate them you realise that she is still there and we’re fighting the eating disorder and she is too. […] And it made it easier, that it wasn’t just her doing this to herself.*

*Emily: I always said, you know, “You’re my daughter, I love you, but anorexia, I can kick as often as I like. If I do something which obviously you don’t like, I do it because it’s against anorexia. It’s not against you, but it is against the illness.”*

*Margaret: I accept that when she was very sick, you know, we talk in these beautiful terms of “it was the eating disorder and not Hayley in the room” and all those euphemisms but effectively Hayley was still in the room but she was treated as though she wasn’t. […] that was probably the most unnerving thing that the person who I believe has been the key to recovery, i.e. herself (emotional tone), was deliberately excluded from the process and talked about as though she wasn’t even in the room.*

*Kristin: […] anorexia never leaves that person. They fight with it every day. […] that disease is so strong!*

Externalisation of the eating disorder enabled Kiera to resist minimising her daughter’s experience to something she was “just doing” to herself and opened the path to position their responses and actions as “fighting the eating disorder”. Externalisation of the illness also enabled these parents to focus their frustration and other negative emotions upon an anorexic entity, rather than their child. For example, externalisation with a battle metaphor enabled Emily to legitimise her strong emotional responses to her daughter’s behaviours—“I love you, but anorexia I can kick as often as I like”. Margaret positioned the practice of externalisation as euphemistic with the effect of Hayley herself also being externalized from the therapy (“deliberately excluded from the process”).

The medical construction of AN as “disease” or “illness” set up an adversarial scenario [50] for these parents where they found themselves engaged in a “fight” to vanquish AN from their daughter’s life. For some of the parents this became a protracted “fight” with the “disease”, for example that led Kristin—after a decade of her daughter struggling with an eating disorder—to conclude that “anorexia never leaves that person”. Michael White [50] has argued that the therapeutic practice of externalisation, when confined to an illness—or other adversarial—metaphor, risks the person (and their family) experiencing a sense of exhaustion, reduced personal agency and increased distancing from others when this does not occur. This was evident in Kristin’s conclusion, “that disease is so strong!” and for Margaret in the inadvertent distancing of her daughter in the externalisation process where she argued that her daughter “was deliberately excluded from the process and talked about though she wasn’t in the room”.

#### “The focus seems to be on all the food aspects”: Impacts of a behavioural focus

The parents’ relief at the MFT/FBT map was followed by a number of their concerns with the narrowing of focus of the MFT/FBT script to their child’s eating behaviour and weight restoration, particularly in the early phases. Although these parents understood the rationale for this symptom focus, they also expressed concerns including the implicit neglect of therapeutic focus on the broader contexts of their child’s distress and the substantive impacts of being allocated the primary role of refeeding their child.

*EXTRACT 4a*

*Kristin: What we’ve realised now is that, because it is a mental disease, so the anorexia, the Maudsley treats the eating part of it. On top of all of that, the big umbrella that’s on top of that is the mental disease that continues for the rest of their days.*

*EXTRACTS 4b*

*Merrum: […] when I said, “we’re at the point now where we need to move on. When’s the psychology part of this come in?” Cause I knew it was going to come. And “oh not yet. We’re not ready for that yet” and I’m saying, but Kate is and I think Kate needs to speak.*

*Susan: Well I think that’s when – I think they were supposed to be but – but it ended up being kind of like problem solving from one week to the next of how to get her to eat. You know you’ve only got an hour and […] her OCD got completely out of control.*

*Margaret: I feel sad that, that she had to, on top of the disease that she is fighting, had to be treated in a way that denied her intelligence, that denied her innate ability to, to fight this herself and of course no parent is going to um, say “oh we’ll just see whether she wants dinner tonight” and not be around.*

These parents expressed concern that MFT/FBT too narrowly treated their daughter’s AN symptoms. Within the narratives of the parents who reported that their child’s weight was restored through MFT/FBT, there was a parallel process between their daughter’s ongoing psychological distress and their own distress and worry that their child might bear the legacy of AN for “the rest of their days” (for example, extract 4a). In the narratives of parents whose child/family discontinued MFT/FBT before weight recovery (extracts 4b), there was significant concern expressed about the exclusion of focus on their child’s broader distress, which parents witnessed through impacts such as their child losing their voice (Merrum) and their child’s unaddressed symptoms of distress (Susan). Implicit in Margaret’s rhetorical positioning was the assumption that parents will do what it takes to get their child well, however, the focus on parents should not exclude the adolescent’s active involvement in their own recovery during any stage of treatment.

*Extracts 5*

*Paul: Though, the focus seems to be all on the food aspects. But from my point of view, like as a parent, the food is the, it’s the end product of the whole problem, what’s going on underneath, what’s causing all this?*

*Terry: […] something causes it in the first instance and not being able to treat what causes it, as well as the anorexia itself, um, trying to separate the two is a problem. And I come back to the need for a more holistic approach […] Something that recognises all the complementary parts and doesn’t try and treat one in isolation of the other.*

In drawing on a problem-solving discourse, Paul argued that there are underlying triggers for his daughter’s experiences—that the eating behaviours are the effect, rather than the cause. Terry’s argument for a “holistic approach” to treatment underscored the fragmented approach to therapy that can occur when mental illness is diagnosed and treated using a nosological framework. The biomedical discourse introduces a tendency to conceive of different expressions of problematic experiences as distinct and separate comorbidities, rather than interrelated parts of a whole. These fathers expressed misgivings about the MFT/FBT scripts, which were increasingly at odds with their own intuitions regarding their daughter’s ongoing needs and struggles.

All of the parents recounted experiences of systemic family distress as they took up the responsibility for their child’s eating restoration.

*Extracts 6*

*Alice: […] they talked to us a lot about the re-feeding and it sounds easy in principle, in theory, but when you actually do it at home it’s not that easy when a teenager’s screaming at the top of her lungs and you’re getting yoghurt thrown at you. I found it really distressing when she didn’t want to eat […] Because you can’t really put someone’s mouth in a vice and make them eat it, can you?*

*Susan: I would say that extreme conflict’s not worth you know. […] if the girl doesn’t think there is any option then that—there’s absolutely no way that you can get out of eating then you’ll eat but you know it’s such a cost.*

*Janice: The Maudsley Method seemed to me, when I was feeling really negative, […]a cheap way out for the government to treat my child, tell us to deal with it. I understand that families are the best approach to deal with this; […] Because you feel a little thrown to the wolves. […] once you close the door of your family home that’s it; you’re on your own.*

*Kristin: You want a live child at the end of it? This is what you have to do. […] but you can only do that with support.*

These parent narratives highlight how the apparent simplicity of the MFT/FBT interventions (“sounds easy in principle”) obscured the reality of how distressing “re-feeding” their adolescent child was for the majority of parents interviewed. This “cost” (Susan) was in navigating the inevitable conflict and dilemmas faced by parents when their child refused to eat. Central to Alice’s rhetorical question, “you can’t really … make them eat it, can you?” is a dilemma related to whether the psychological risks of forcing a child to eat can be justified in the name of saving their life.

The costs of these interventions were also a private cost for parents, and were positioned by Janice as “a cheap way out for the government to treat my child”. This statement exposes the broader institutional investments in MFT/FBT to treat adolescent AN that are obscured by the uptake of parental discourses that assume that a “good” parent will unquestionably assume the role of “re-feeding”. Taking up this parental role unquestionably (“you want a live child at the end of it?”—Kristin) obscured both the burden on these parents and families and the institutional positioning of MFT/FBT as a cost-effective treatment for adolescent AN [51].

### Theme 2—Negotiating guilt and responsibility

All of the parents struggled to negotiate guilt related to their daughter’s experience of AN and its treatment. The practice of allocation of responsibility for the “refeeding” of their child also held the potential to induce further guilt, particularly in contexts where anticipated weight gains were not achieved or maintained.

#### Navigating responsibility and guilt in relation to etiology

All the parents engaged in processes of negotiating the question of guilt related to the etiology of AN, taking up a diverse range of positions within a parent-blaming discourse.

*Extracts 7*

*Terry: […] we’d failed as parents. It’s simple, you know? Parents are supposed to look after their kids and particularly in, in the traditional sense fathers are supposed to look after their families. […] I felt that I’d failed in that.*

*Jack: […] my role as provider, protector and nurturer it felt very, very fundamental that um, you know the basics of parenthood I failed.*

*Janice: I felt that we didn’t deserve it. I felt that we’d been—this is going to sound terribly, um, ah, I can’t think of the right terminology—I felt this happened to other people that weren’t as good parents as we were. […] I was knocked off my ivory tower. So I was very, very angry. [...] At the time I just, yeah, I thought I’d really failed as a parent to—to have sort of allowed this to happen, or not noticed this had happened. But it wasn’t until the psychiatrist we first saw and he said the same to me, was that genetics loads the gun and the environment fires it. […] that this wasn’t my fault at all.*

*Emily: […] you feel like well, what have I done? Well, for me as a mother it felt like, well as a parent, what have we done wrong. How (pauses) how did it go off the rails? Did we not pay enough attention? […] But she still says, “Mum, you will not let me put on that much weight again.” We have a good relationship and, you know, she doesn’t say it to hurt me, but for me, that then triggers again, oh it was my fault.*

Although eight parents talked about how the MFT/FBT therapists had explicitly absolved them of guilt, the majority of the parents’ narratives continued to be positioned by parent-blaming discourses. This ranged from guilt about the cause, not noticing their child’s symptoms and intervening sooner, and a sense of somehow having failed their child. In taking up a father-as-protector discourse, Terry and Jack assumed their child’s AN experience to be a neglect of their responsibilities (“supposed to look after their families”). Janice simultaneously took up and rejected the parent-blaming discourse with her anger being understood as an identity violation and in defense of herself and her husband as “good parents”. Despite seeking to free herself from this discourse, she nevertheless remained entangled in the assumption that AN arises from faulty parenting. Emily was caught in a position where she felt responsible and guilty for not discerning AN earlier, responsible for the “refeeding” process, and her daughter’s weight gain-related distress. These extracts highlight that the guilt experienced by parents was complex and multifaceted, and not readily amenable to straightforward anti-blame rhetoric.

#### Allocation of responsibility for refeeding

All of the parents expressed how they struggled with self-blame in the context of being allocated responsibility for their adolescent’s eating restoration.

*Extracts 8*

*Alice: Yeah, and when things aren’t going well you do kind of blame yourself. Like I really blame myself for her going backwards at Christmas. I should have been more watchful about what had happened …*

*Emily: My daughter said, “That dietician saved me from dying because she made me eat.” […] She accepts people with knowledge, and so she goes like right, this lady is a specialist, she is a very no nonsense specialist, and my daughter accepted that that’s the way she is, and she goes, “Okay. I will do what that lady tells me.”*

Alice experienced guilt when her daughter’s weight dropped and blamed herself for not continuing the hypervigilance around her daughter’s eating. Here it is evident that although the MFT/FBT script maintains that parents are not responsible for AN, being allocated responsibility for refeeding places parents in a position where they are directly responsible for weight recovery, and therefore recruited into further guilt when this is not achieved or maintained. By insisting on the inclusion of a dietician in the treating team, Emily was able to free herself from the position of being solely responsible for her daughter’s eating, which reduced conflict within her family. For Margaret and Jack, whose daughter was unable to gain weight with the MFT/FBT intervention, the protracted struggles experienced by their daughter to gain weight resulted in the purportedly non-blaming intervention having a paradoxical effect (extracts 9).

*Extracts 9*

*Margaret: […] because it was ground hog day. […] it turned into this sort of circular um, blaming is probably going too far, but a sort of circular evaluation of, of where we’d gone wrong, because the framework clearly works so if we’ve not succeeded,* ***we’ve*** *gone wrong. […] (Interviewer: How did that affect how you saw yourself as a mother?) Oh my goodness I was, I have never been so challenged in my life as a mother. I felt wretched. It’s such a, a fundamental thing to feed and protect your child and anorexia has already challenged that in its essence and then to, on a weekly basis, be in a context (emotional tone to voice) where you’re failings are on show and also on show to your children.*

*Jack: Well no in our case we were on trial. I’d be shocked if Margaret didn’t even use that expression. […] I felt like she was on trial in those sessions towards the end and Hayley’s failure to put on weight was Margaret’s failure to feed her enough ultimately. And I didn’t come to her defense, either in the room or afterwards, you know, well enough or consistently enough.*

The experience of “groundhog day” with MFT/FBT built on the already established sense of falling short of the “fundamental thing to feed and protect your child” for Margaret. The ongoing allocation of responsibility for Hayley’s eating restoration to Margaret and Jack and the search for ways to get Hayley to eat created a context for the allocation of blame—“**we’d** gone wrong”. For Margaret, this contributed to an erosion of her sense of identity as a mother (“I felt wretched”). Jack also expressed concern about how this non-blaming approach, when protracted, also become mother blaming.

### Theme 3—Navigating uncertainty

For these parents, the reallocation of responsibility to their daughters for eating and self-care, and their transition to autonomy in the later phases of MFT/FBT, were troubling to negotiate, especially in the context of their child’s ongoing psychological distress and/or ED symptomology.

#### Fear and struggles with shifting roles

Around half the parents expressed fear that their child’s struggles might continue for the rest of their lives.

*Extracts 10*

*Jane: […] she’s always, gets upset easy and could get depressed easily, you think there’s so many more years to go, you know. How’s her life going to go? Is she going to constantly live with being a bit depressed, or not?*

*Kristin: Yeah, and it will never go and I think, for me, that bothers me [emotional voice] because she’s my daughter, you know?*

*Paul: I think it’s the not knowing that is the hardest of it. I mean, if you have a flu or a cold, you know you’re going to get over it. […] That it’s got a set a time for you to recover from it …*

*Terry: I think we’re all realistic enough to know that once you’ve suffered anorexia, it’s probably something that will be there in the background for the rest of your life...*

These parents questioned whether their child’s psychological distress is an experience they will “constantly live with” (Jane) and “will never go” (Kristin), as these parents sought to renegotiate their relationship with hope for their child’s future. Paul’s struggle with uncertainty about his daughter’s recovery highlighted the limitations of applying a medical discourse to AN [52], as recovery from AN is not as simple or certain as recovery from a “flu or a cold”. For Terry, the diagnosis of AN entailed suffering “that will be there in the background for the rest of your life”. His use of “your” signifies that the suffering was not only for his daughter but also for his family, because for parents there is also the prospect of lifelong distress as they seek to support their child.

Six parents’ narratives highlighted their struggles in re-allocating responsibility to their child for their eating in light of their ongoing fears.

*Extracts 11*

*Emily: I actually have difficulty then again letting go, because it was then, okay I have to be in charge to be really the boss against this illness. […] Kelly said to me, “Mum, don’t talk about it anymore. I don’t want to be that person with anorexia all the time. We have to let it go.” […] it’s hard to not identify then [as] the mother of a child with anorexia.*

*Jane: To some extent you just have to say, “Well look, what else can I do?” Just what will be will be, to a little extent. […] it’s the same as you can lead a horse to water but you can’t make them drink and they’re growing up and you have to let them go eventually.*

*Kiera: I think that’s part of the Maudsley that when they’re younger that you do the parenting thing and make the decisions and take over, which is what you do best, and last year when she was 19 that still needed to be the case because she wasn’t able to do that for herself. […] I told her when she was younger, I had to look into her eyes and tell her that dad and I would be her strength until she was able to be stronger for herself [sobs].*

A turning point for Emily in rethinking her role as “the boss against this illness” and renegotiating her identity as “the mother of a child with anorexia” was when her daughter resisted totalisation of her identity as “that person with anorexia”. Jane was also faced with knowing that she had done everything she could as a mother—which she depicted through metaphor (“lead a horse to water”)—to mark out the limitations of the extent to which parents can make their child eat, particularly as they get older and to allow for uncertainty to “a little extent”. Kiera talked about the necessity of taking responsibility for her daughter’s eating when she lapsed at age 19 as she sought to continue to be strong for her daughter with the hope that one day she will have the “strength” to do this for herself. Shifting from an authoritarian stance in phase one of the intervention was troubling for these parents as they were faced with the fear of their daughter lapsing, greater uncertainty around their child’s future, and renegotiating their roles and identities as parents.

### Where do we turn now?

For the four parents whose daughters did not gain weight with MFT/FBT, there was a sense of helplessness and fear that the front-line, evidence-based treatment did not work for their child.

*Extracts 12*

*Susan: One of them [treatment team] said basically they were running out of options, I mean they—we had kind of—they did come up with lots of options but we were kind of scraping the bottom of the barrel.*

*Margaret: I think it was a combination of um, of fear of, what else, if not this? We’d also had a period where we really did feel Hayley needed hospitalisation […] and in spite of her statistics, they clearly weren’t ‘bad’ enough to be hospitalised and we weren’t prioritised enough. So I think I’d felt absolutely abandoned by the system.*

*Merrum: I’m not an expert but I could tell that my child was ready for the next part and they weren’t listening. […] But I just felt like her voice wasn’t being heard and it was so important for her. As soon as kids verbalise things, they own it.*

For Susan, not gaining weight after numerous hospitalizations and MFT/FBT created a parallel process of helplessness through the family system and her treatment team. Margaret and her husband Jack experienced a sense of “abandonment” as they fell through the cracks of a treatment system that was unable to be tailored to their daughter’s unique situation. Merrum struggled to negotiate her identity as a mother with the sense of herself as “not an expert” in AN treatments. Nevertheless, her refusal to sideline her expertise to the treatment team led her to draw on her insider knowledge as a mother that prioritised her daughter’s voice in her recovery.

All parents whose child’s weight was restored with MFT/FBT expressed uncertainty and unease regarding the ongoing eating-related and psychological distress that their daughters were experiencing, and a sense of struggle about where to turn to next.

*Extracts 13*

*Kiera: […] we should have had her seeing a psychologist [post-treatment] just to cope with the stress of things. […] because we just thought that she was cured. A bit naïve with all the stuff that we know now...*

*Paul: It’s like a virus, changing to another—the treatment works for the initial virus but it doesn’t work for the new virus now. […] Whereas now we have more of an understanding but we still feel quite helpless in it, because we don’t really know what to do. I mean, apart from helping our daughter get some more therapy, we don’t really know what else we can do.*

In the context of their child’s ongoing difficulties, these parents sought to understand how it was that their child had not recovered. After discharge from treatment, Kiera reported being surprised to learn that it is not uncommon for AN to have a much longer course, leaving her with the retrospective understanding of herself as “naïve”. Paul expanded upon the medical discourse through likening his daughter’s ongoing struggles to a “virus” which mutates out of their control. A dualistic medical discourse of illness and cure failed to provide a trajectory beyond recovery or chronic illness, leaving these parents feeling “helpless” and struggling to know where to turn next.

These parents struggled to know where to turn to next, particularly as MFT/FBT had been positioned to many of them—through the certainty of scientific discourse—as the only “evidence-based” treatment for adolescent AN. They were positioned on the outside of this discourse, in a place of uncertainty about the next step and how to stand for their child in the face of ongoing ED symptoms and psychological distress.

## Discussion

The structure and promise of MFT/FBT were initially experienced as ameliorating these parents’ anxieties about their child’s struggle with AN. Although the intervention went some way towards addressing their guilt through non-blaming rhetoric and the practice of externalisation of the illness, these parents’ complex relationship with guilt—particularly when coupled with their child’s ongoing ED symptoms and psychological distress—meant that this non-blaming approach paradoxically became experienced as parent-blaming. Being allocated responsibility for their child’s eating in the early phases of treatment was also troubling for these parents. In particular, they expressed frustrations with the focus on weight gain and eating behaviours at the expense of psychological processes and, in some instances, their child’s loss of voice. When they completed or discontinued MFT/FBT they were faced with an uncertain future in relation to their child and were unsure where to turn in the face of their child’s ongoing ED symptomatology and/or psychological distress.

### Behavioural focus of MFT/FBT

Most parents in this study expressed concerns with the extent of the behavioural focus and emphasis on weight and eating restoration, particularly in the early phases of MFT/FBT. This was particularly distressing for parents whose child did not gain weight, with the concomitant increased systemic interpersonal conflict and heightened self-blame. These parents’ experiences are echoed by a parent interviewed in an Australian Broadcasting Commission report on AN treatments, where one mother whose child later ended her own life said: “One psychiatrist was giving us advice about what we should do and I said I didn’t know if I could do this, hold Tess down and put food in her mouth without hurting her,” she said. “He said I needed to go into therapy so I could find out why it was that I couldn’t save my daughter’s life.” (see news article at the following link: https://www.abc.net.au/news/2017-05-04/australian-health-system-failing-patients-with-eating-disorders/8485300\).

All parents whose child initially regained weight were also troubled by their child’s recurrent psychological distress post-treatment, particularly depression and ED symptoms, leaving these parents without a map to navigate their responsibilities for their child’s recovery. Although there is evidence that early weight gain is a predictor of improved psychological outcomes [53], AN is commonly associated with a range of other mental health problems [54, 55]. Additionally, it is notable that from the parents’ perspectives, their daughter’s concerns about body weight and shape were not ameliorated by MFT/FBT—despite weight gain—which is consistent with the findings of other research [56]. Given the difficulty of treating AN—and the great distress of the families involved—it is understandable that clinicians need to prioritise the adolescent’s medical safety. However, these parents expressed clear concern about the apparent limitations of MFT/FBT in addressing their child’s comorbid psychological issues—whether pre-existing or concomitant with AN. Early treatment focus on nutritional restoration with the assumption that this will also reduce an adolescent’s distress is problematic, as an absence of comprehensive intervention for adolescent and parent systemic distress in the early phases of treatment means that these issues are effectively relegated until the later phases of therapy. As argued by Greg Dring, “if the therapist spends the first sixteen sessions of the work discouraging the discussion of feelings, relationship issues and developmental difficulties in a personal way, then it may be very difficult to revive such discussion at a later stage when, in any case, the work is about to be concluded.” ([57], p. 66).

### Parental guilt and the etiological stance of MFT/FBT

The agnostic etiological stance of MFT/FBT and the explicit absolving of guilt and blame was experienced as helpful by most parents in this study. However, since the treatment involves a focus on modifying parenting practices, it remains an open question as to what role parenting styles and family dynamics play in the development or maintenance of AN, noting also that a recent study has found that parent-focused treatment was more effective than FBT [15]. Most of these parents harboured lingering guilt and regret about their roles in potentially influencing and/or struggling to discern the onset or relapse of AN. Parents also sought to understand the causal factors associated with their child’s experience of AN through the uptake of the dominant medico-scientific discourse, in which deterministic causation is a fundamental feature.

Despite the agnostic etiological stance of MFT/FBT, these parents took up a wide range of etiological positions, which underscored the uniqueness of each family’s experiences and understandings of AN. This included: an innate, organic, disease model; a genetics and environment model; a sociocultural model; an emotional coping model; a controlling mothers model; a confluence of factors model; and a self-confidence and control model. MFT/FBT attempts to pragmatically sidestep these considerations with its agnostic stance, however this also created significant frustration in parents who argued that underlying or associated psychological difficulties were sidelined by the intervention.

In the context of having a child with significant health issues, feelings of guilt and responsibility are inevitable within the prevailing dominant parenting discourses. Despite the anti-blame rhetoric of MFT/FBT, these parents continued to be both positioned and troubled by parenthood discourses that hold parents responsible for their child’s distress and difficulties. The experiences of persistent and residual guilt expressed by these parents support the view that MFT/FBT does not adequately address guilt and responsibility in a family-systemic manner [57]. Instead, parents were left to understand and interpret their child’s experiences and actions, and therapeutic opportunities were therefore lost to re-ascribe meaning to guilt, including the possibility of (re)authoring their identity narratives [45] through what might be absent but implicit in guilt [58], such as a valuing of their responsibility in the parental role.

### Externalisation

Externalisation is a feature of NT [13], and it has been adopted by MFT/FBT to externalise the illness [7], with the intention of therapeutically shifting parental blame and criticism to an externalised entity. When externalisation is confined to a dualistic biomedical discourse, an adolescent’s experience is constructed as an illness/disease that is totalised as bad, with recovery—as the antithesis of illness/disease—constructed as entirely good [59, 60]. Michael White [50] has argued that dualistic conceptions of AN set up the task of therapy as adversarial and risk leading to a sense of exhaustion when this does not eventuate. Instead relational, rather than dualistic, externalisation [52] has been proposed in which the adolescent’s voice is at the centre of a therapeutic conversation that maps the real effects of AN on their life and identity, fostering the generation of their own experience-near metaphors [45] to richly depict both their AN experience and fuel the building of counter-narratives that depict their processes of re-covering their life and identity [52] from AN.

### Beyond MFT/FBT

When MFT/FBT is positioned to parents as an effective, gold-standard treatment within a biomedical discourse, parents are recruited into an understanding that there is a likelihood that their children will be “cured” of their “disease”. This risks a sense of hopelessness and helplessness when the treatment does not lead to the elimination of AN. Parents in this study expressed fears for their child’s futures as they negotiated the transition to independence and adulthood, in the presence of ongoing psychological distress. Even more concerning for these parents was the apparent understanding that once MFT/FBT failed, their daughter’s life trajectory would inevitably be one filled with chronic mental health issues. What was evident in parental narratives was their commitment to their children, and some of the ways that they sought to preserve their identities as parents, within a therapeutic context where there was limited opportunity for them to re-author durable and sustaining identities for themselves and their child.

### Clinical implications

This study has questioned the core tenets of MFT/FBT, including the practices of allocating responsibility to parents for re-feeding and weight restoration and the deferring of addressing in detail an adolescent’s distress until the final phase of treatment. Additionally, the practice of externalisation of the illness is limited in its effectiveness at reducing blame and responsibility to parents, particularly in contexts where the child does not progress on to recovery. The central metaphor of illness in externalisation also risks excluding the adolescent, who may not conceptualise their experiences in the terms of this discourse [52]. It also constructs the task of therapy within an adversarial framework, resulting in a sense of exhaustion if recovery does not eventuate [50].

This study highlights the need for further research into the development of more comprehensive treatments for AN. It also highlights the importance of therapists tailoring treatments to a person and family’s needs and preferences, which is a critical—and sometimes neglected—facet of evidence-based practice [61]. In particular, it is clear that parents want and need treatments that address the range of issues related to their child’s difficulties from the outset of treatment—treatments that are not rigidly focused upon eating behaviours and weight gain at the exclusion of addressing other psychological problems within the broader context of their lives.

### Research scope

This study was interested in exploring the experiences of a group of parents who were concerned to improve the first-line treatment for adolescent AN, including those who were currently experiencing or had discontinued this therapy. The children of these parents continued to experience psychological distress during and post-treatment; with some continuing to experience ED symptomatology. More research is needed into the diverse experience of parents who experience MFT/FBT along with their adolescent child and siblings, and this research represents one facet of the complex array of possible experiences. Although the themes that were constructed from the data were present across the parent narratives, future research with larger sample sizes might consider variables such as time post-MFT/FBT intervention, the age and gender of the adolescent, parental separation, the extent of sibling involvement in treatment, and the inter-relationships between parent, adolescent and sibling experiences of MFT/FBT.

## Conclusions

Parent’s experiences of their child’s struggles with AN are complex. MFT/FBT interventions respond to this complexity through an early focus on eating and weight restoration. The substantive risk of this approach, particularly when applied as a first-line treatment for all adolescents who experience AN, is the potential to inadequately address the psychological distress of adolescents and their families, particularly in the early phases of treatment. Since MFT/FBT interventions are family-based, and harness parents as the primary treatment providers, it is crucial that parents’ experiences and voices are heard and inform the development of improved treatments for AN. These parents have highlighted the need for therapeutic interventions to be mindful of the unintended effects of allocating responsibility to parents for re-feeding their adolescent, and to more comprehensively address their child’s distress (particularly in the earlier phases), as well as parental guilt, blame, and exhaustion from efforts to fight and vanquish AN. The findings of this study invite practitioners and researchers to consider therapeutic interventions that move beyond a behavioural focus on AN symptoms, including in the earlier phases of treatment—interventions that more holistically address the impacts of AN on the life and identity of the adolescent and their family and that provide a space for parents to re-author their stories as stories of standing for their child in hope, and as stories of survival.

## Appendix A

### Interview Schedule

A selection of questions will be used with each participant and questions will scaffold between

- Experience (e.g. Can you tell me about …?)
- Meaning (What does … mean to you?)
- Identity (e.g. How does … have you seeing yourself as a person, what does this say to you about what matters to you as a parent/what is important to you/what you value?)
- Positioning on experience/identity conclusion (e.g. is this OK for you or not? Why?)

A) **Interview will be augmented with a timeline for narrative/discourse**

1. Can you tell me your story of anorexia as a family? OR When did you notice anorexia starting to have an influence over your child’s life?
2. (PROMPT) What was this like for you as parents?
3. (PROMPT) Do you consider your child’s relationship with anorexia to be problematic for them now? If so, how? If not, why?
4. How have these experiences affected yourself as a person? As a mother/father/parent?
5. What does your child having experienced anorexia mean to you as a parent? Is this helpful or unhelpful? Why?
6. How have your lives changed with your child’s experience of anorexia*? What have you got in touch with yourselves as a person/parent?

B) **EXPERIENCES OF MAUDSLEY/FAMILY-BASED THERAPY (FBT)**

1. How did you come to be involved in Maudsley family-based therapy (FBT)?
2. What have been your experiences of FBT as parents?
3. What was most helpful about FBT? Why?
4. What was least helpful about FBT? Why?
5. Did you complete the Maudsley FBT? Why or why not?
6. Did FBT assist you to shift your child’s relationship with anorexia?

**Ask (C)** **OR** **(D) depending on FBT status:**

C) **If the adolescent/family completed FBT (IF NOT ANSWERED WITH OPEN QUESTIONS ABOVE)**

1. When did your family complete FBT? What has your child’s relationship with anorexia been like since you completed FBT?
2. How has participating in the Maudsley FBT affected how you see yourself as a person? Parent? Is this helpful or not? Why?
3. Overall, what stands out for you if you reflect on what impacts FBT had on you as a parent? Was this helpful or not? Why?
4. Do you have any fears of ceasing M-FBT?

D) **If the adolescent/family discontinued FBT**

1. Can you tell us about the circumstances/your experiences that led your family to no longer continue with FBT?
2. What has your child’s relationship with anorexia been like since then? How has this been for you as a person and as parents since discontinuing FBT?
3. Looking back how do you make sense of why you chose as parents not to continue with FBT?
4. Are you settled with the decision or not? Why?
5. How did participating in the Maudsley FBT affect how you see yourself as a person? Parent? Is this helpful or not? Why?
6. Did you have any fears/concerns about what might happen if you continued or discontinued FBT?
7. What were you hoping for as you made the decision to cease FBT?

E) **Questions to all participants regardless of whether completed or discontinued FBT**

1. What do you want to not forget about yourself/your child/your family from the Maudsley FBT?
2. What might be important for another family to know as they commence FBT?
3. What advice might you give to a person or family who is about to start FBT?
4. What would you like more of in future treatments for anorexia? What would you like less of in future treatments for anorexia nervosa? What does this say to you about what matters for you/your child/your family?
5. Do you have hopes for future treatment/therapy in terms of the sort of work you would like to engage in as a family? What does this say to you about what you value as a person/parent?
6. Has our conversation today been helpful or unhelpful or both? Why?
7. What has stood out for you from our conversation today?
8. What might be important for us not to forget as we analyse the data from this interview?

**PROMPTS:**

**The following questions will be asked about particular aspects of Maudsley/FBT if participants do not cover these areas from the above open lines of questioning.**

**Externalisation of anorexia as an illness**

1. Did you experience externalisation of AN as an illness? What do you understand by this? Was it helpful or not? Why?

**Family meal and parental instruction at family meals**

1. Did you experience a family meal with the therapist in the room? If so, what was this like for you?
2. Did you feel that the therapy worked with your expertise as a parent?
3. Did you ever find that your strategies differed from your partners? Or from the approach? What was this like for you? How did you respond?
4. Were you ever asked to take a position on the anorexia? What was this like? Helpful or not? Why?

**Parental Unity**

1. Did you experience parental unity as part of the process of Maudsley?
2. Was parental unity hard at times? How did you respond? How was this responded to in therapy? Helpful or not? Why?
3. Did you ever see another therapist during M-FBT?

## Appendix B

### Parent member check feedback (participant pseudonyms)

**Susan:** […] I am so grateful that you have done this. I think the themes that have emerged from your research are extremely valid. Even my daughter would say (and I am sure I told you this) “why aren’t they treating my psychological problems?”. I can’t read it too closely today because it makes me cry (again) but generally I am very happy with the way my views have been represented.

**Paul:** I have read the report and it is very accurate. It is a great report. There is a lot of emotion in those sentences from parents, and many common feelings. I think it will help a lot to understand what is going on in families. I wish you all well with the report, and hope it helps future families.

**Janice:** the analysis of my interview transcript reflects my thoughts and experiences accurately. [...] with the benefit of some years hindsight now I still feel very much the same way that the Maudsley method, whilst largely effective for our child, did hinge almost entirely upon our ability to put (at least one) of the parents lives on hold to be able to effectively implement the therapy recommended. Also with the benefit of hindsight, (anecdotally) we have come to believe that the younger the child at diagnosis and the personality type of that child also has a huge impact on the effectiveness of treatment.

**Terry:** Thanks for the copy of your draft paper. I have no additional comments or amendments.

**Margaret and Jack**: We are very comfortable with you using the extracts and the related conclusions. We found the study all the more compelling for the parallel experiences that you have recounted and the recommendations you have made.

**Emily:** Reading through the transcript brought back a lot of memories. I had to stop a couple of times and then recommence. I think it pretty much tells it how it is: not enough medical care put into the underlying issues (ED is certainly just a “tool” to help the affected person with their mental health issues—control).

**Alice:** I am happy with how my comments were interpreted and also how these comments were used in the article. Thanks for the opportunity to contribute. I think it’s a very important paper.

## Appendix C

### Researcher Positioning Statements

**Ella Wufong**: As someone entering the field of clinical psychology with a rich and varied background, it is apparent to me that psychological rigidity is at the core of most mental health issues, and that flexibility and responsiveness are two key features of effective therapy. A Skinnerian conception of evidence-based practice is limited in scope when results are restricted to blunt outcome measures, and the evaluation of therapeutic models on this basis runs the risk of engendering orthodoxy and reductionism, limiting the development and availability of therapeutic alternatives. I believe that a partial antidote to this is research that prioritises the voices and experiences of the people who seek therapy, or have therapy imposed upon them. Furthermore, I strongly believe that it is vitally important for clinical research to also consider sociocultural and historical contexts, and to explicitly examine and question philosophical and conceptual assumptions at every turn.

**Janet Conti:** My background in the field of eating disorders was initially as a dietitian in the 1990s, then as a Clinical Psychologist and now a Senior Lecturer in Clinical Psychology. Throughout this time, I have sought to prioritise the voice of the experiencing person in my clinical work, including in narrative therapy, and research. This research arose out of conversations with Paul Rhodes and a number of health professionals who shared my concerns for a group of people in Australia who have been termed by some as “the Maudsley refugees”, signifying a gap in services for those whom the first-line treatment for adolescent AN does not work. This research is one arm of a larger research project that is aimed to give voice to adolescents, parents and the clinicians who treat them about their experience of MFT/FBT in an effort to expand therapeutic options available to these adolescents and their families.

**Paul Rhodes:** My background is in the field of family therapy, having been a practitioner and leader in the Australian New Zealand Journal of Family Therapy for many years. I have now been pursuing research in a wide range of fields, including critical psychology, community-based approaches to emotional distress, art-based research and others. For a long period in the 2000’s I served as a Team Leader in the family-based treatment of eating disorders, developing services in Sydney and Australia. Now, nearly 20 years on I have come to see some difficulties with the model, despite still seeing its value as a clinical practice.

## Abbreviations

AN: Anorexia nervosa
APS: American Psychological Society
ED: Eating disorder
FBT: Family-based therapy
IBW: Ideal body weight
MFT: Maudsley family therapy
NT: Narrative Therapy
PFT: Parent-focused therapy

## Footnotes

1 Despite some divergence between the intervention models over time [57], the terms MFT and FBT are often used interchangeably, and they will be referred to as MFT/FBT throughout this paper.
